# Exercise-Induced Circulating Irisin Level Is Correlated with Improved Cardiac Function in Rats

**DOI:** 10.3390/ijerph17113863

**Published:** 2020-05-29

**Authors:** Dae Yun Seo, Jun Hyun Bae, Tae Nyun Kim, Hyo-Bum Kwak, Pham Trong Kha, Jin Han

**Affiliations:** 1 National Research Laboratory for Mitochondrial Signaling, Department of Physiology, BK21 Plus Project Team, College of Medicine, Cardiovascular and Metabolic Disease Center, Inje University, Busan 47392, Korea; sdy925@gmail.com (D.Y.S.); kimtn031@gmail.com (T.N.K.); phamtrongkha@hus.edu.vn (P.T.K.); 2 Smart Marine Therapeutics Center, Inje Univeristy, Busan 47392, Korea; 3 Institute of Sport Science, Seoul National University, Seoul 08826, Korea; baexx068@snu.ac.kr; 4 Department of Kinesiology, Inha University, Incheon 22212, Korea; kwakhb@inha.ac.kr

**Keywords:** irisin, exercise, cardiac function, pro-inflammatory cytokines

## Abstract

Irisin, a recently identified myokine, plays an important physiological role in modulating energy homeostasis. However, the role of irisin in cardiac function during exercise has not been evaluated. In this study, we investigated the effect of exercise on irisin, pro-inflammatory cytokines, and cardiac function during 12 weeks of exercise in rats. Eight-week-old Sprague-Dawley male rats were randomly divided into two groups (n = 9 per group): sedentary control (CON) and exercise (EXE) groups. The EXE group was trained on a motorized treadmill at 20 m/min, for 60 min/day, five times/week for 12 weeks. The EXE group showed a decrease in abdominal visceral fat (*p* < 0.05), epididymal fat (*p* < 0.01), and total cholesterol (TC) (*p* < 0.05) and an increase in irisin levels (*p* < 0.01). Irisin negatively correlated with abdominal visceral (*p* < 0.05) and epididymal fat (*p* < 0.05) and positively correlated with the ejection fraction (*p* < 0.05), fractional shortening (*p* < 0.05), and cardiac output (*p* < 0.05). In conclusion, exercise decreases the abdominal visceral and epididymal fat and TC levels, possibly caused by elevated irisin levels, thus improving the cardiac function. This suggests that exercise-induced circulating irisin levels correlate with improved cardiac function in rats.

## 1. Introduction

Exercise can prevent a number of chronic diseases [1,2,3]. During exercise, the contraction of skeletal muscles releases molecules called myokines [4]. Myokines are involved in the autocrine regulation of metabolism in the muscles, adipose tissue, liver, and brain [5]. One such myokine, irisin, is cleaved and secreted from a type I membrane protein called fibronectin type III domain-containing protein 5 (FNDC5) [6]. Irisin is predominantly released from the skeletal muscles and is regulated by increased expression of a receptor activated by peroxisome proliferator-activated receptor-γ coactivator 1α (PGC-1α) to stimulate intracellular mitochondrial biogenesis (e.g., mitochondrial uncoupling protein mRNA 1 (UCP1)) [7,8] and phosphorylation of p38 mitogen-activated protein kinase (p38 MAPK) [9]. Irisin increases energy expenditure, which promotes weight loss and improves insulin resistance [6]. Moreover, irisin has a positive influence on metabolic syndromes and insulin resistance [10]. Circulating irisin has been shown to improve the clinical course of acute coronary syndrome [11] and to promote umbilical vein endothelial proliferation [12] through the ERK signaling pathway [9,13].

Irisin is released from the skeletal muscle [4] and adipose tissue [14,15] during exercise. In addition, irisin can be a predictive biomarker in patients with acute heart failure [16]. This thermogenic adipomyokine produced by FNDC5 is known to have a potential role in preventing obesity and metabolic syndrome [3]. A study showed that lower irisin concentrations were associated with lower 12-month survival rates in patients with coronary artery disease [11]. Additionally, in obese patients who were put on an 8-week hypocaloric diet, increasing irisin plasma levels were associated with enhanced insulin sensitivity [17]. Compared to people with normal weight, obese patients had higher circulating irisin levels, which also correlated with higher body weight (kg), body mass index (BMI: kg/m^2^), fat mass (kg), and fat free mass (kg) [18]. Irisin affected insulin metabolism and obesity-related factors.

Additionally, irisin is positively associated with BMI and adiposity [17,18]. Similarly, irisin is related to glucose and lipid homeostasis in patients with obesity and metabolic syndrome [19]. In normal healthy animals, a C57/BL6 mouse treated with irisin (100 mg/kg) showed remarkable ventricular functional recovery, and irisin treatment reduced the size of the myocardial infarct via increasing SOD-1 and p38 phosphorylation. Irisin induced cardio-protection and improved mitochondrial function [20]. Irisin levels are closely related to cardiovascular disease and metabolic syndrome. Previous studies have focused on the relationship of irisin with obesity and its cardiovascular clinical implications. However, the direct effect of irisin on cardiac function after exercise remains unclear. 

The prevailing challenges among patients with metabolic syndrome are high fat mass, high blood cholesterol levels, and secondary diseases including cardiovascular disease, leading to high mortality [21,22]. Numerous previous studies have shown that exercise-induced irisin affects the fat metabolism [6,7,14,19] and cardiac function, thereby decreasing the risk of cardiovascular disease [11,16,20,23,24]. The underlying mechanism for this effect is that exercise-induced irisin level increases the mRNA expression of UCP1 in the adipose tissue [25]. A recent study found that irisin is significantly associated with fat metabolism; higher irisin levels resulted in lower fat mass [26]. However, further studies are necessary to elucidate the relationship between irisin and exercise, and the mechanism through which it lowers the fat mass by acting as an intermediate mediator. The current evidence in normal, healthy adults showed differences in irisin levels (high level of irisin after exercise) after maximal exercise workload [27]. Moreover, eight weeks of resistance exercise increased irisin levels in obese adults (BMI > 23 kg/m^2^) [28]. In an animal model, the cardiac muscle produced more irisin than the skeletal muscles following aerobic exercise [29]. Hence, irisin has been linked to cardiovascular disease and fat metabolism. Although it is well-known that exercise actively boosts the cardiovascular function and impacts the lipid metabolism, the exact relationship between irisin as an intermediate mediator and myokines expressed during exercise remains to be elucidated. 

We believe that exercise-induced increased irisin levels stimulate cardiac function. Furthermore, it has been predicted that increased irisin level can reduce the blood lipid levels and fat mass. Therefore, the objective of this study was to quantify the time-dependent irisin levels and determine the functional link between irisin and cardiac function, fat mass, and blood lipid levels in rats during 12 weeks of exercise. 

## 2. Materials and Methods 

### 2.1. Animals

Eight-week old Sprague-Dawley male rats were randomly divided into two groups (n = 9 per groups): sedentary control (CON) and exercise (EXE) groups. They were housed in cages, with two rats per cage. All rats were given ad libitum access to standard rat chow and water, and were housed in an environment with controlled temperature (23 ± 2 °C) and a relative humidity of 40% under a 12:12-h light-dark cycle. The study was approved and performed according to the guidelines of the Inje Medical University Animal Care and Use Committee [30].

### 2.2. Exercise Protocols

Rats in the EXE group exercised on a treadmill (Columbus Instruments, Columbus, OH, USA) for one week. After the adaptive exercise, rats were trained five times per week; each training session lasted approximately 60 min at a speed of 20 m/min and a grade of 0% for 12 weeks [31]. This treadmill training is considered a moderate-intensity exercise for rats [32]. 

### 2.3. Blood and Tissues Preparation 

After 12 weeks of exercise, all rats were anesthetized with sodium pentobarbital (60 mg/kg body weight), blood samples were collected, and the body and dissected heart were weighed. Blood samples obtained from the heart were centrifuged at 1977× *g* for 30 min at 4 °C to separate the plasma and were then stored at −80 °C until further analysis. The heart was immediately frozen in liquid nitrogen and stored at −80 °C for protein extraction.

### 2.4. Biochemical Analysis

The levels of total cholesterol (TC), high-density lipoprotein cholesterol (HDL-C), low-density lipoprotein cholesterol (LDL-C), and triglycerides (TG) were measured using an automated blood analyzer (Hitachi 7600-210 and 7180, Tokyo, Japan) via a colorimetric assay based on enzymatic reaction. Glucose level was measured using an automated glucose analyzer (ADVIA 1650, Bayer). Insulin (Millipore, Corp., Billerica, MA, USA) and irisin were measured using enzyme-linked immunosorbent assay kits, (Millipore, Corp., Billerica, MA, USA) and (Phoenix Pharmaceuticals, Inc., Burlingame, CA, USA), respectively. Tumor necrosis factor (TNF)-α, interleukin (IL)-6, high-sensitivity C-reactive protein (hs-CRP), and matrix metalloproteinase (MMP)-9 were measured using enzyme-linked immunosorbent assay kits from R&D systems (Company, Minneapolis, MN, USA). All biochemical analyses were performed according to the manufacturers’ instructions. 

### 2.5. Echocardiography 

Twenty-four hours before the final exercise session, an examination was performed using a VIVID 7 Dimension system (General Electric-Vingmed Ultrasound, Horton, Norway) with a 5.5–12 MHz transducer on a heated platform to monitor vital signs. To perform transthoracic echocardiography, rats were anesthetized with isoflurane inhalation with 2% isoflurane. After anesthetization, rats were positioned on the heated platform and allowed to inhale isoflurane enriched with oxygen at a flow of 5 L/min. Two-dimensional M-mode images were recorded in the center of the left ventricle (LV). We obtained the following M-mode parameters: LV mass, left ventricular internal dimension end-diastole (LVIDd) and systole (LVIDs), LV ejection fraction, interventricular septum thickness end-diastole (IVSd) and systole (IVSs), fractional shortening and left ventricular posterior wall thickness end-diastole (LVPWd) and systole (LVPWs) [31]. 

### 2.6. Statistics

Data are presented as means (SD) (standard deviation). To determine data normality, we used the Shapiro–Wilk test (*p* > 0.05). The Mann–Whitney test analyzed the differences between the rats in the CON and EXE groups. The correlation with irisin level, fat, and cardiac function was determined using the Spearman (r*_s_*) method. All data were considered significant at *p* < 0.05. 

## 3. Results 

### 3.1. Basic Characteristics between the Control and Exercise Groups after 12 Weeks of Exercise 

Table 1 presents the body, heart, abdominal visceral fat, and epididymal fat weight after 12 weeks of exercise. There was a 5% decrease in the body weight of rats in the EXE group compared to that of rats in the CON group; however, the levels did not show any significant difference. The respective weights of the heart (10%, *p* < 0.05), abdominal visceral fat (54%, *p* < 0.05), and epididymal fat (63%, *p* < 0.01) were significantly lower in rats in the EXE group than in those in the CON group. 

### 3.2. Comparison of Lipid Profiles between the Control and Exercise Group after 12 Weeks of Exercise

Table 2 presents the lipid profiles in the CON and EXE groups after 12 weeks of exercise. The TC levels showed a 12% decrease in the EXE group compared to those in the CON group (*p* < 0.05). The HDL-C, LDL-C, TG, glucose, and insulin levels did not significantly differ between the two groups.

### 3.3. Comparison of the Levels of Irisin and pro-Inflammatory Cytokines between the Control and Exercise Groups

Table 3 presents the irisin, TNF-α, IL-6, hs-CRP, and MMP9 levels after 12 weeks of exercise. There was a 46% increase in the irisin levels of the EXE group compared to that of the CON group (*p* < 0.01). The TNF-α, IL-6, hs-CRP, and MMP9 levels were not significantly different between the two groups. 

### 3.4. Comparison of Echocardiography Variables between the Control and Exercise Groups

Table 4 presents the M-mode and B-mode data after 12 weeks of exercise. There was an increase in the ejection fraction (12%, *p* < 0.05), fractional shortening (17%, *p* < 0.05), cardiac output (29%, *p* < 0.01), stroke volume (21%, *p* < 0.05), end-diastole volume (34%, *p* < 0.05), and end-systolic volume (58%, *p* < 0.05) in the EXE group compared to those of the CON group. The IVSd, IVSs, LVIDd, LVIDs, LVPWd, LVPWs, and heart rate were not significantly different between the two groups. 

### 3.5. Correlation between Irisin Level and Abdominal Visceral Fat and Epididymal Fat 

**Figure 1:**
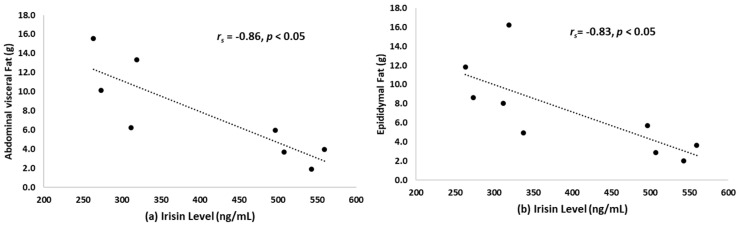
(**a**) The correlation between abdominal visceral fat (g) and irisin level; (**b**) the correlation between epididymal fat (g) and irisin level.

Figure 1 presents the negative correlation between abdominal visceral fat R_s_(8) = −0.86, *p* < 0.05) and epididymal fat R_s_(9)= −0.83, *p* < 0.05 with irisin level. 

### 3.6. Correlation between Irisin Level and Ejection Fraction (EF), Fractional Shortening (FS), Stroke Volume, and Cardiac Output

**Figure 2:**
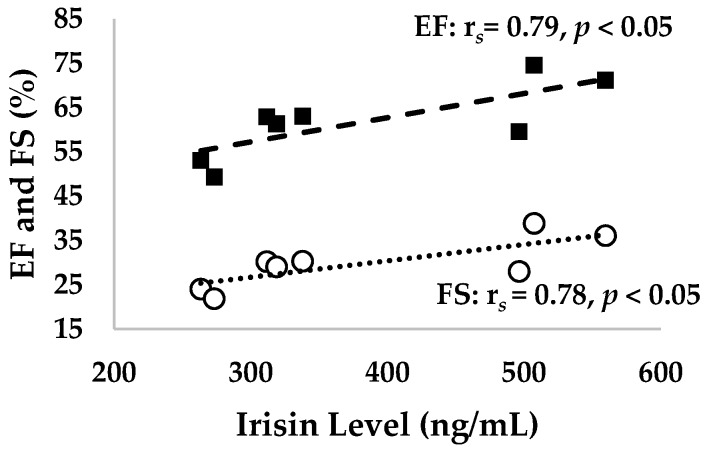
The correlation between the irisin levels and ejection fraction (%) as well as fractional shortening (%).

**Figure 3:**
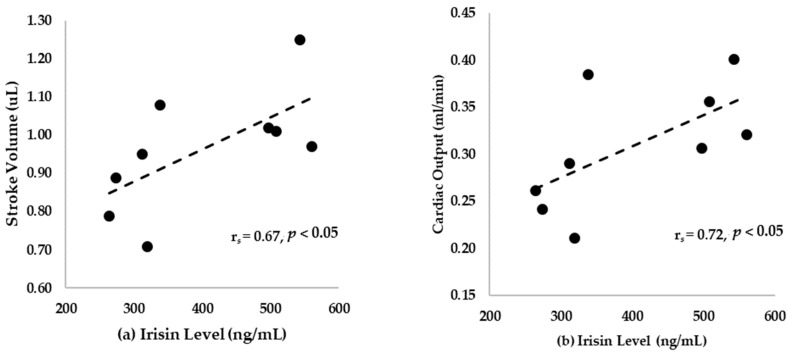
(**a**) The correlation between stroke volume (µL) and irisin level; (**b**) the correlation between cardiac output (mL/min) and irisin levels.

Figure 2 and Figure 3 present the correlation of irisin levels with EF (r*_s_* = 0.79, *p* < 0.05), FS (r*_s_* = 0.78, *p* < 0.05), stroke volume (r*_s_* = 0.67, *p* < 0.05), and cardiac output (r*_s_* = 0.72, *p* < 0.05) in cardiac function. 

## 4. Discussion

In the present study, we found that TNF-α, IL-6, hs-CRP, and MMP9 levels did not significantly differ between the two groups. The exercise group showed a decrease in abdominal visceral and epididymal fat and the TC levels, and improved cardiac function with an increase in irisin levels. Furthermore, irisin levels negatively correlated with the abdominal visceral and epididymal fat and positively correlated the EF, FS, and cardiac output. To the best of our knowledge, this is the first study that correlates exercise-induced circulating irisin level with improved cardiac function in rats. 

In our study, exercise increased the irisin levels and decreased the TC levels (Table 2 and Table 3). These results are similar to a previous study, which showed the effect of the irisin in lowering the TC levels in the plasma of patients with type 2 diabetes [33]. Although the EXE group in this study did not have type 2 diabetes, our results support the findings of previous studies that reported improved lipid metabolism due to an increase in irisin. Moreover, another study showed that the body fat mass and TC decreased in normal, healthy male rats after eight weeks of exercise, compared to rats fed with a high-fat diet. This indicates that exercise improved these parameters through irisin production [34]. Hence, this study indicates that irisin-induced increased acetyl CoA carboxylase-β phosphorylation in skeletal muscle and UCP1 expression in the fat cells and decreases fat weight and serum TC levels, thereby affecting TC levels alone through exercise [35]. Moreover, the exercise-induced AMPK signaling pathway attenuated the effect of irisin on glucose uptake and fatty acid β-oxidation in myocytes [36]. Overall, our findings support the hypothesis that exercise-induced irisin can modulate fat mass and lipid profiles. 

Our findings also indicate that irisin is negatively related to abdominal visceral and epididymal fat mass (Figure 1). This result supports the hypothesis that endurance exercise leads to an increase in irisin levels and decrease in abdominal visceral fat [37]. It also shows that irisin is associated with lipid parameters [38]. Therefore, the abdominal visceral and epididymal fat were associated with the overexpression of 11 β-hydroxysteroid dehydrogenase type 1 (11-β HSD-1) in the adipose tissue [39]. In addition, obese mice fed a high-fat diet underwent a change in FNDC5 protein expression in the adipose tissue via exercise-induced irisin, which affected the browning of adipose cells [40]. Similarly, exercise-induced irisin promoted FNDC5 gene expression to increase the fat cell metabolism, and improved glucose metabolism and insulin sensitivity [41]. This evidence is related to irisin’s pleiotropic role in improving the muscle and adipocyte metabolism [42]. In the present study, glucose and insulin levels showed a slight tendency to decrease with exercise; however, changes did not reach statistical significance. This might support the hypothesis that irisin influences the single nucleotide polymorphisms in FNDC5 changed glucose metabolism and insulin sensitivity in normal healthy models [41,42,43,44].

Moreover, irisin has a direct anti-inflammatory role by preventing the pro-inflammatory activation of adipocytes [45]. However, our findings show that the levels of TNF-α, IL-6, CRP, and MMP9 did not improve in the exercise group. This could be because the rat models used in our study were neither obese nor had metabolic syndrome. However, previous studies reported that irisin attenuated pro-inflammatory cytokines in subjects with obesity, and metabolic syndrome and improved inflammation in the adipose tissue [46]. Increased irisin levels may have reduced TNF-α and IL-6 by reversing the effect of lipopolysaccharide [45]; this would confirm that irisin exerts anti-inflammatory effect via inhibiting the expression and function of pro-inflammatory cytokines TNF- α and IL-6 [45]. In addition, TNF-α is correlated with insulin resistance [47]. IL-6 also prevents non-oxidative glucose metabolism and decreases lipoprotein lipase leading to an increase in the plasma triglycerides levels [48]. These expressions were not significantly different between the control and exercise groups in this study; only TC showed a decrease in the exercise group. Nevertheless, the mechanisms of effects of irisin on inflammation in obesity and metabolic syndrome are yet to be elucidated. 

In our study, a 12-week exercise showed a positive correlation between irisin and cardiac functions such as EF and FS (Table 4 and Figure 2). Our results also show improved cardiac output, stroke volume, diastolic volume, and systolic volume after exercise, which were correlated with irisin levels. The results validate previous findings where irisin was shown to increase myocardial contractility by improving intracellular Ca^2+^ and mediating p38-MAPK and the ERK pathway [49]. This also supports previous findings that irisin mitigated pathologic cardiac hypertrophy inducing effect of autophagy pathway via AMPK-ULK1 signaling [50]. In addition, our findings support a previous study which showed that the release of myokine irisin improved the post-MI (myocardial infarction) remodeling and LV systolic function by the downregulation of β-MHC and increasing α-SMA expression [51]. Another possible explanation for this result is that exercise increased the irisin level and improved it through PGC-1α activation. Elevated levels of exercise-induced irisin prevented cardiovascular disease and reduced cardiac fibrosis by improving the Nkx2.5^+^ in cardiac progenitor cells (CPC) to repair cardiac fibrosis [3]. Specifically, irisin upregulated Ki67 of proliferative markers and phosphorylated histone 3 and reduced histone deacetylase 4 to increase p38 in CPC [52]. These results suggest that exercise-induced irisin can improve Nkx2.5+ cells to protect against cardiovascular damage and improve cardiac function via increasing PGC-1α expression [53]. Furthermore, our results support the previous findings that a high level of irisin is negatively associated with cardiovascular complications [23]. Our results also validate previous findings that patients with heart failure had high levels of circulating irisin and this correlated with metabolic parameters and the index of oxidative stress [54]. We aimed to determine the potential effect of irisin on cardiac function after exercise. The results of this study show that exercise-induced irisin does affect cardiac function as well as abdominal visceral and epididymal fat mass. Exercise was found to have a beneficial effect in decreasing the fat mass and TC. This study also showed the adipokine and myokine effect of irisin. Overall, irisin can improve the fat mass and lipid profiles, thereby improving the cardiac function. 

This study has some limitations. First, although the statistical power effect size was at 0.35 and the power was at 0.8 for all statistical tests, the sample size was small. Second, the animal model used in this study was not related to the metabolic syndrome model, most previous studies showed the effects of irisin in metabolic syndrome models. In addition, this study did not consider sex differences to examine the effect of physiological differences on exercise. Furthermore, although this study showed an improvement in cardiac function, we could not provide representative echocardiography images. Finally, our study cannot comprehensively explain the mechanisms underlying irisin-mediated cardiac function improvement after exercise training. Further studies are necessary to understand the mechanisms between irisin and cardiac function in detail. 

## 5. Conclusions

Our study found that exercise resulted in a decrease in abdominal visceral fat and epididymal fat and TC levels along with an increase in irisin level and improvement in cardiac function. Furthermore, irisin was negatively correlated with abdominal visceral and epididymal fat and positively correlated with the EF, FS, and cardiac output. To the best of our knowledge, this is the first study that studies the correlation between exercise-induced circulating irisin level and improved cardiac function in rats.

## Figures and Tables

**Table 1:** Comparison of characteristics between the control and exercise group.

Parameters Mean (SD)	CON (n = 9)	EXE (n = 9)	z	*p*-Value
Body weight (g)	542.88 (44.61)	512.67 (49.76)	1.28	0.20
Heart weight (g)	1.33 (0.13)	1.19 (0.08)	2.06	0.04
Abdominal visceral fat weight (g)	7.99 (4.67)	3.60 (1.50)	2.06	0.04
Epididymal fat weight (g)	8.98 (3.86)	3.27 (1.33)	3.14	0.002

CON: control group, EXE: exercise group.

**Table 2:** Comparison of lipid profiles between the control and exercise group.

Parameters Mean (SD)	CON (n = 9)	EXE (n = 9)	z	*p*-Value
TC (mg/dL)	55.11 (6.94)	48.00 (6.83)	2.07	0.04
HDL-C (mg/dL)	45.11 (5.69)	38.88 (6.49)	1.88	0.06
LDL-C (mg/dL)	15.89 (4.81)	14.44 (3.43)	0.80	0.42
TG (mg/dL)	37.22 (5.33)	34.44 (5.94)	1.33	0.18
Glucose (mg/dL)	129.89 (23.98)	110.25 (6.14)	1.69	0.09
Insulin (ng/mL)	0.38 (0.37)	0.22 (0.05)	1.11	0.27

CON: control group, EXE: exercise group, TC: total cholesterol, HDL-C: high-density lipoprotein cholesterol, LDL-C: low-density lipoprotein cholesterol, TG: triglycerides.

**Table 3:** Description of the irisin and pro-inflammatory cytokines levels between the control and exercise group.

Parameters Mean (SD)	CON (n = 9)	EXE (n = 9)	z	*p*-Value
Irisin (ng/mL)	332.64 (94.52)	486.78 (101.64)	−2.21	0.003
TNF-α (pg/mL)	22.84 (1.59)	25.67 (1.03)	−1.29	0.20
IL-6 (pg/mL)	16.35 (0.89)	17.19 (1.03)	−1.88	0.06
hs-CRP (μg/mL)	0.4 (0.6)	0.19 (0.03)	1.37	0.17
MMP9 (ng/mL)	1.82 (0.39)	1.81 (0.45)	0.27	0.79

CON: control group, EXE: exercise group, TNF-α: tumor necrosis factor, IL-6: interleukin-6, hs-CRP: high-sensitivity C-reactive protein, MMP9: matrix metalloproteinase-9.

**Table 4:** Description of the echocardiogram variables between the control and exercise group.

Parameters Mean (SD)	CON (n = 9)	EXE (n = 9)	z	*p*-Value
M-Mode				
IVSd (mm)	1.59 (0.18)	1.60 (0.12)	−0.40	0.69
IVSs (mm)	2.29 (0.25)	2.44 (0.28)	−0.58	0.56
LVIDd (mm)	8.80 (0.56)	9.15 (0.88)	−0.71	0.45
LVIDs (mm)	6.47 (0.69)	6.40 (1.05)	0.49	0.63
LVPWd (mm)	1.89 (0.30)	1.92 (0.20)	−0.50	0.62
LVPWs (mm)	2.36 (0.22)	2.22 (0.37)	1.29	0.20
Ejection fraction (%)	57.20 (6.42)	64.32 (6.82)	−2.12	0.03
Fractional shortening (%)	26.62 (3.81)	31.37 (4.70)	−2.12	0.03
LV Mass (g)	1.53 (0.14)	1.65 (0.13)	−1.84	0.07
B-Mode				
Heart Rate (bpm)	327.39 (36.31)	349.90 (43.03)	−1.11	0.23
Cardiac output (ml/min)	0.27 (0.03)	0.35 (0.04)	−3.09	0.002
Stroke volume (uL)	0.83 (0.11)	1.01 (0.17)	−2.30	0.02
End-Diastolic volume (uL)	1.32 (0.17)	1.77 (0.44)	−2.38	0.02
End-Systolic volume (uL)	0.53 (0.10)	0.84 (0.27)	−2.38	0.02

CON: control group, EXE: exercise group. LV: left ventricle, IVSd: interventricular septum thickness end-diastole, IVSs: interventricular septum thickness end-systole, LVIDd: left ventricular internal dimension end-diastole, LVIDs: left ventricular internal dimension end-systole, LVPWd: fractional shortening and left ventricular posterior wall thickness end-diastole, LVPWs: fractional shortening and left ventricular posterior wall thickness end-systole.
